# Design and Characterization of Elastic Artificial Skin Containing Adenosine-Loaded Solid Lipid Nanoparticles for Treating Wrinkles

**DOI:** 10.3390/pharmaceutics13010033

**Published:** 2020-12-28

**Authors:** Sooho Yeo, Sukkyun Jung, Heui Kyoung Cho, Young Ho Kim, Gi Hwan Kim, Dohyun Kim, Byoung Hyen Ko, Jaehwi Lee

**Affiliations:** 1 College of Pharmacy, Chung-Ang University, Seoul 06974, Korea; sooho32@hanmail.net (S.Y.); jsk0314@cau.ac.kr (S.J.); dylan13@naver.com (D.K.); badol95@naver.com (B.H.K.); 2 R&D Center, Megacos, 16, Simin-daero 327 Rd, Dongan-gu, Anyang-si, Gyeonggi-do 14055, Korea; hkcho1901@megacoskorea.com (H.K.C.); yhkim1609@megacoskorea.com (Y.H.K.); ghkim1612@megacoskorea.com (G.H.K.)

**Keywords:** adenosine, solid lipid nanoparticles, elastic artificial skin, anti-wrinkle, skin permeation

## Abstract

Adenosine (AD), which is used for treating wrinkles, exhibits poor skin permeation. The aim of the present study was to develop a cross-linked silicone-based cellulose elastomer as an elastic artificial skin for the treatment of skin wrinkles, a biocompatible lipid-based nano-carrier for enhancing the skin permeation of AD, and a formulation consisting of the lipid-based carrier incorporated in the elastic artificial skin. AD-loaded solid lipid nanoparticles (SLNs) were prepared using a double-emulsion method. Particle characteristics and mechanical properties of SLNs and elastic artificial skin, respectively, were assessed. Skin permeation was evaluated using SkinEthic RHE tissue, a reconstructed human epidermis model. The mean particle size and zeta potential for SLNs ranged from 123.57 to 248.90 nm and −13.23 to −41.23 mV, respectively. The components of neither SLNs nor the elastic artificial skin were cytotoxic, according to cell- and tissue-viability assays and EU classification. SLNs and the elastic artificial skin exhibited sustained drug release for 48 h. The amount of AD released from SLNs and elastic artificial skin was approximately 10 times and 5 times higher, respectively, than that from AD solution. Therefore, elastic artificial skin incorporated with AD-loaded SLNs may serve as a promising topical delivery system for cosmeceutical treatment of skin wrinkles.

## 1. Introduction

Cosmeceuticals are cosmetic pharmaceutical hybrids formulated as topical drug delivery systems that improve the appearance of the skin by enhancing its health and beauty [1,2,3]. Products that are designed to treat aging or dried skin, including anti-wrinkle agents, show promising potential in the skin-care market. Wrinkles, an indication of skin aging, are attributed to loss of skin function by decreasing collagen in the skin, which is caused by aging of fibroblasts as collagen synthesizing region [4,5,6]. In addition, a decrease in collagen results in reduced skin elasticity.

Based on the studies of skin wrinkles, some of the materials were stimulating fibroblast growth. Adenosine (AD) (molecular weight, 267.24 g/mol; adenine-9-β-d-ribofuranoside) is an active pharmaceutical ingredient for the treatment of skin wrinkles [6,7,8,9]. It exerts its pharmacological effect through the A_2A_ receptors, which are one of the AD related receptors in cells and stimulate collagen production from primary human dermal fibroblasts [6,10]. An increase in the amount of collagen promotes dermal wound healing, as well as wrinkle reduction [6,11].

Despite its promising pharmacological effects, AD exhibits poor permeation into the skin owing to its hydrophilic property [10,11]. Various approaches have been reported to improve permeation of hydrophilic drugs into skin such as inclusion of chemical enhancers (such as ethanol and dimethyl sulfoxide) [12,13], physical methods utilizing an instrument (such as electroporation and iontophoresis) [14,15,16], and optimized formulations (such as polymer particles, emulsion, and lipid-based particles) [17,18,19]. However, considering safety issues associated with the disruption of the skin barrier and the investment in specialized facilities, pharmaceutical alternatives are necessary as an alternative to complicated technologies [20,21,22,23].

Adenosine has been delivered by liposomes, lipid emulsions as well as solid lipid nanoparticles (SLN) [24,25,26]. SLN systems effectively improve the permeation of hydrophilic drugs into the skin with relatively low skin irritation with high loading capacity compared with liposome and emulsion, and production cost [27,28]. In SLN systems, a hydrophilic drug is encapsulated in a lipid matrix via preparation of a water-in-oil-in-water (W/O/W) phase [29,30]. In addition, organic solvents are not used when preparing SLNs owing to their bio-toxicity [30,31]. The success of a topical drug delivery system depends on the ability of the drug to permeate the stratum corneum and the maintenance of sufficient drug concentration in the epidermis [32]. In this regard, the nano size and lipid components of SLNs facilitate the permeation and retention of drugs into the skin [19,32]. The small particle size (less than 500 nm) and the W/O/W phase of lipid-based particles are particularly advantageous for drug permeation because the stratum corneum is composed of corneocytes layers alternated to intercellular lipid organized in lamellae; therefore, the affinity between lipid-based particles and the epidermis is enhanced [19,29,33,34]. In addition, after application, SLNs can form a thin lipid membrane on the skin surface, which has been reported to prevent trans-epidermal water loss that facilitates stratum corneum hydration and enable sustained release of the encapsulated drugs [19,32,35].

Researchers continue to search for a cosmeceutical formulation that is closer to the ideal for treating skin wrinkles. Artificial skin systems composed of polymers have broad applications in biomedical engineering [36,37,38]. The term “artificial skin” first appeared in Yang et al.’s paper to treat wrinkles [39]. Several types of bioengineered skin have been developed in the cosmetic industry, for wound dressing, and as drug delivery carrier systems [40,41,42]. Commercially available systems are primarily classified into two categories: Carrier systems prepared using natural biomaterials such as chitin, chitosan, and hyaluronic acid, and those prepared using synthetic polymer materials such as polydimethyl siloxane [43,44,45]. Synthetic materials exhibit unique characteristics such as high elasticity, easy availability, and good biocompatibility [46,47,48]. Among synthetic materials, silicone elastomers are the most promising in terms of low toxicity, good biocompatibility, physiological inertness, excellent thermal and oxidative stability, and removability [49,50,51,52]. For anti-wrinkle treatment, Yu et al. recently designed a cross-linked elastic artificial skin film based on a two-component polysiloxane consisting of a W/O emulsion of vinyl/hydrogen dimethicone and a catalyst. Upon application to the skin and formation of the silicone-based elastic artificial skin film, physical and skin adhesive forces pull the applied skin, which results in reduction of wrinkles [53]. Elastic artificial skin is a highly convenient topical system because it can be quickly formulated via a cross-linking reaction by mixing two-components of A (vinyl dimethicone + Karstedt catalyst) and B (hydrogen dimethicone) [49,52,54].

In the present study, we prepared elastic artificial skins containing AD-encapsulated SLNs for the treatment of skin wrinkles. AD was loaded into SLNs to improve its permeation into the skin. Moreover, we evaluated the particle characteristics and mechanical properties of SLNs and elastic artificial skin, respectively. Regarding biological assay, previous studies have evaluated skin irritation and permeation on rat skin. However, special attention is contended to be given to possible improvements in animal welfare. Moreover, EU Cosmetics Directive has prescribed the use of alternative methods to animal testing [55,56,57]. Among the various models of reconstructed human epidermis, which mimic the biochemical and physiological characteristics of the human epidermis, the SkinEthic RHE model presents a histological morphology analogous to in vivo human tissue [56,58]. Therefore, in this study, we evaluated skin irritation and permeation using the SkinEthic RHE tissue model.

## 2. Materials and Methods

### 2.1. Materials

We purchased AD, potassium dihydrogen phosphate (potassium phosphate monobasic, KH_2_PO_4_), isopropanol, sodium dodecyl sulfate (SDS), thiazolyl blue tetrazolium bromide (MTT), niacinamide, glycerin, edetate disodium, 1,2-hexanediol, and 1,3-propanediol from Sigma-Aldrich (St. Louis, MO, USA); lauric acid, myristic acid, palmitic acid, and stearic acid (SA) from SAMCHUN (Pyeongtaek, Korea); glycerol monostearate from Kanto Chemical Co. Inc. (Tokyo, Japan); poloxamer (PX) 188 and PX 407 from BASF (Ludwigshafen, Germany); Span^®^ 80 (SP 80), Span^®^ 40 (SP 40), Span^®^ 20 (SP 20), and Tween^®^ 80 from Dae Jung Co. Ltd. (Busan, Korea); and HRC-LS-2830/1A and HRC-LS-2830/1B from HRS Co. Ltd. (Seoul, Korea). Cellulose nanofiber-graft-vinyltrimethoxysilane was a generous gift from Korea Institute of Industrial Technology (KITECH, Incheon, Korea). Serasense SF1 was purchased from KCC Basildon Chemicals Ltd. (Abingdon, UK); DC^®^ RM 2051 from Dow Corning (Midland, MI, USA); M-Hydro EG from INKOS Co. LTD. (Incheon, Korea); Sepimax Zen and Sepiplus 400 from SEPPIC (Paris, France); Aristoflex AVC from Clariant (Muttenz, Switzerland); Olivem 1000 and Olivem 900 from HallStar (Arcore, Italy); phosphate-buffered saline (PBS) from Tech & Innovation (Chuncheon, Korea); Dulbecco’s modified eagle medium (DMEM) from WelGENE (Gyeongsan, Korea); and Roswell Park Memorial Institute (RPMI) 1640 medium, Dulbecco’s PBS (DPBS), fetal bovine serum (FBS), and penicillin-streptomycin solution 100× from BioWest (Nuaillé, France). Fibroblast cell lines (CCD 986sk and Detroit 551) were purchased from the Korea Cell Line Bank (Seoul, Korea) and SkinEthic RHE tissue from SkinEthic Laboratories (Nice, France). Methanol of high-performance liquid chromatography (HPLC) grade was purchased from Honeywell (Seelze, Germany). All other chemicals utilized were of HPLC grade.

### 2.2. Preparation of AD-Loaded SLNs

AD-loaded SLNs were prepared using a modified double-emulsion method (W_1_/O/W_2_) via a hot high-pressure homogenization (HPH) technique. Briefly, the oil phase containing SA and SP 80 was melted at 10 °C above the lipid melting point. The inner aqueous phase containing AD was poured into the molten oil phase and homogenized using Ultra-Turrax T18 (IKA, Staufen, Germany) at 5000 rpm, which resulted in a primary W_1_/O emulsion. Then, the W_1_/O/W_2_ nano-emulsion was prepared via a two-step homogenization method, that is, high-speed homogenization and HPH. The primary W_1_/O emulsion was added to the hot aqueous surfactant solution (W_2_) consisting of PX 188 and homogenized using Ultra-Turrax T18 at 1000 rpm. To fabricate nanoparticles, the W_1_/O/W_2_ emulsion was homogenized in a NanoDeBEE homogenizer (BEE international, South Easton, MA, USA) for 3 cycles under 20,000 psi. The nano-sized emulsion was rapidly cooled using an ice bath and stored under 4 °C for further use. To evaluate the effects of different compositions of AD-loaded SLNs, different SLN compositions were assessed, as presented in Table 1. Additionally, the effect of various HPH parameters (homogenization pressure and number of homogenization cycles) were evaluated, as presented in Table 2.

### 2.3. Synthesis of Elastic Artificial Skin

The composition of the elastic artificial skin is shown in Table 3 and Table 4. The elastic artificial skin was prepared by dissolving HRC-LS-2830/1A in Serasense SF1, which formed component A, and HRC-LS-2830/1B, Sepimax Zen, and Aristoflex AVC in distilled water (DI water), which formed component B. Thereafter, each remaining ingredients were added to components A and B, respectively, and completely dissolved until the mixture was transparent and a viscous gel was obtained. The two-compositions should be separated during storage to prevent cross-link reaction prior to using them. When applying the elastic artificial skin compositions to the reconstructed skin models, the components A and B were mixed in a ratio of 7:3 to react cross-link reaction.

### 2.4. Preparation of Elastic Artificial Skin Containing AD-Loaded SLNs

The elastic artificial skin containing AD-loaded SLNs was prepared by adding AD-loaded SLNs to component B at proportions indicated in Table 5 and homogenized using a high-speed homogenizer at 10,000 rpm. The prepared elastic artificial skins incorporated with AD-loaded SLNs were stored in a refrigerator at 4 °C to eliminate bubbles.

### 2.5. Characterization of Ad-Loaded SLNs

#### 2.5.1. Determination of Nanoparticle Size, Polydispersity Index (PDI), and Zeta Potential

Particle size and PDI of the prepared SLNs were determined at 25 °C via dynamic light scattering using Zetasizer Nano ZS (Malvern instruments Ltd., Worcestershire, Malvern, UK). The samples were diluted 10 times with DI water before measurement. For particle size, the instrument was equilibrated prior to every measurement. Each value reported was the average of three measurements.

The zeta potential of the SLN was measured at 25 °C using Zetasizer Nano ZS and estimated from the electrophoretic mobility of the particle surface. The samples were diluted 10 times with DI water before measurement. The instrument was equilibrated prior to every measurement. Each measurement was performed in triplicate.

#### 2.5.2. Determination of Drug-Loading Capacity

The loading efficiency (LE) and loading amount (LA) of AD-loaded SLNs were determined using centrifuge (Gyrozen 1580 MGR, Gyrozen Ltd., Daejeon, Korea). SLN preparations were diluted 10 times with DI water to a final volume of 2 mL and then gently vortexed. The suspension was then centrifuged at 14,000 rpm, 4 °C for 2 h. The free drug concentration in the supernatant was analyzed using HPLC, as described in Section 2.8. LE and LA were calculated from Equations (1) and (2), respectively.

(1) $$\mathrm{LE}\;\left(\%\right)\;=\;\frac{\mathrm{Mass}\;\mathrm{of}\;\mathrm{encapsulated}\;\mathrm{drug}\;}{\mathrm{Mass}\;\mathrm{of}\;\mathrm{drug}\;\mathrm{initially}\;\mathrm{added}}\;\times \;100$$

(2) $$\mathrm{LA}\;\left(\%\right)\;=\;\frac{\mathrm{Mass}\;\mathrm{of}\;\mathrm{encapsulated}\;\mathrm{drug}}{\mathrm{Mass}\;\mathrm{of}\;\mathrm{nanoparticles}\;}\;\times \;100$$

### 2.6. Mechanical Properties of the Elastic Artificial Skin

#### 2.6.1. Viscosity Behaviors of Synthesized Elastic Artificial Skin

The rotational rheometer (HAAKETM Rheostress 1, Thermo Fisher Scientific Inc., Karlsruhe, Germany) was operated with P 35 Ti L geometry and MPC 35 plate for measuring zero-shear viscosity. Before measurement, the equilibrium condition was maintained for 10 min at an interval of 1 mm and a shear stress of 0.01 Pa. The zero-shear viscosity was determined at a shear stress of 5 Pa and ambient temperature.

#### 2.6.2. Thickness Evaluation of Elastic Artificial Skin

The artificial skin film was formed using a rheometer to control the spread force to apply. Two milliliters of A and B were carefully placed on a stage of the rheometer and P 35 Ti L geometry was applied until intimate contact. The rheometer was operated for 20 min at an interval of 1 mm and a shear stress of 10 Pa. We evaluated the average thickness of one film by measuring the thickness of the formed elastic artificial skin at three different selected locations using a digimatic caliper (Mitutoyo, Tokyo, Japan). The data were represented as the means ± standard deviations (SD) of three replicates measurements.

#### 2.6.3. Tensile Strength (TS), Elongation at Break (EAB), and Young’s Modulus (YM)

TS, EAB, and YM of the elastic artificial skins were determined using a texture analyzer (Stable Microsystem, TA.XT Plus, Godalming, Surrey, UK), according to the method described by Hazirah et al. [59] and Schmid [60] with slight modification. Briefly, elastic artificial skins from each film formulation were cut into 1 cm × 5 cm strips that were placed onto grip pairs of AT/G probe, which were then attached to the texture analyzer with a 5 kg load cell. The initial gap was set to 50 mm. The elastic artificial skin strips were subsequently stretched by moving the upper grip at a head speed of 1 mm/s until the elastic artificial skin broke.

TS was calculated using Equation (3). F_max_ is the maximum load (N) needed to pull the sample apart, and A is the cross-sectional area (mm^2^) of the film sample.

(3) $$\mathrm{TS}\;\left(\mathrm{MPa}\right)=\frac{F_{\max}\;\left(N\right)}{A\;\left({\mathrm{mm}}^{2}\right)}$$

EAB was calculated from Equation (4), where L_max_ is the film elongation (mm) at the moment of rupture, and L_0_ is the initial grip length (mm) of the sample.

(4) $$\mathrm{EAB}\;\left(\%\right)=\frac{L_{\max}}{L_{0}}\;\times \;100$$

YM was calculated from Equation (5). Stress is the load (N) divided by the area (mm^2^), and strain is the change in length (mm) divided by the original length (mm).

(5) $$\mathrm{YM}\;\left(\mathrm{MPa}\right)=\frac{\mathrm{Stress}\;\left(\mathrm{MPa}\right)}{\mathrm{Strain}}$$

### 2.7. In Vitro AD-Release Studies

An in vitro AD release study was performed using the dialysis bag method. Dialysis bags, with a molecular weight cut-off of 12–14 kDa (Spectrum Laboratories, Inc., Compton, CA, USA), were soaked in deionized water for 12 h prior to the experiment. The initial amount of AD in SLN was obtained based on the loading amount values. Initially 1 mL of SLN dispersions (equivalent to 10 mg of lipid) was placed into the dialysis bag, and both ends were sealed using a string. Dialysis bags were then immersed in 70 mL vials containing 50 mL of PBS (pH 7.4) as release medium, according to the method described by Pritchard et al. with modification [61]. The vials were then placed in a shaking water bath (BS-21, LAB companion, Seoul, Korea) and shaken at 70 rpm and 37 ± 0.5 °C. At predetermined time intervals (1, 2, 4, 8, 12, 24, and 48 h), aliquots of 1 mL were withdrawn from the vial, passed through 0.45-μm membrane filters (PTFE Syringe Filters, Membrane-solutions, Plano, TX, USA), and immediately analyzed using HPLC, as described in Section 2.8.

### 2.8. HPLC Analysis

The concentration of AD was determined using a Waters HPLC with the Breeze 2 analysis program (Waters, Milford, MA, USA) with a CapCell Pak C_18_ MG column (5 µm, 4.6 mm × 250 mm Shiseido, Tokyo, Japan) at ambient temperature. We detected AD by measuring its absorbance at 260 nm using a UV–Visible spectrophotometer with a Waters 2487 Dual λ Absorbance Detector (Waters, Milford, MA, USA). The mobile phase was prepared by mixing 0.05 M KH_2_PO_4_ buffer and methanol at a ratio of 85:15 (*v/v*), filtering the solution through a 0.45-μm nylon membrane filter, and subsequently degassing it in a sonicator for 10 min. The mobile phase was allowed to pass at a flow rate of 1.0 mL/min. The composition of the mobile phase was selected based on the proper separation of AD and appropriate retention time. The injection volume was 10 µL. A calibration curve was constructed for AD. The exact amount of 4 mg of adenosine powder was added to 100 mL of mobile phase and completely dissolved. Then the solution was serially diluted with the mobile phase to prepare five standard solutions with concentrations of 0.5–8 µg/mL. Each adenosine standard solution was injected into HPLC after passing through 0.45-μm PTFE membrane filters (R^2^ = 0.9999).

### 2.9. In Vitro Irritation Studies

#### 2.9.1. Irritation Study Using Skin Fibroblast Cell Lines

To evaluate skin irritation, we investigated the cytotoxic effects of each component of the nanoparticles in human fibroblast cell lines. Two fibroblast cell lines (CCD 986sk and Detroit 551) were seeded into 96-well plates at 1 × 10^4^–1 × 10^6^ cells/well, respectively; the number of cells were calculated using the hemocytometer method. Prior to each cytotoxicity experiment, the cells were incubated to adhere for 24 h at 37 ± 0.5 °C in a humidified atmosphere with 5% CO_2_. Then, the incubated cells were exposed to the samples at 20 µL/well for 15 min at room temperature. The negative control (NC) and positive control (PC) used were DPBS and 5% SDS solution, respectively, according to the method described by Alépée et al. [55]. The exposed cells were subsequently rinsed with sterile PBS. The treated cells were incubated for 24 h at 37 ± 0.5 °C, 5% CO_2_ in 200 µL of growth medium for the MTT reduction experiment, as described in Section 2.9.3.

#### 2.9.2. Irritation Study Using SkinEthic RHE Tissue Model

Upon receipt of the SkinEthic RHE tissues, which had been grown in a 24-well plate format, culture inserts were removed from the nutrient gel and transferred under aseptic conditions into a new sterile 6-well plate containing 1 mL of a growth medium provided by the manufacturer. The tissues were incubated at 37 ± 0.5 °C in a humidified atmosphere with 5% CO_2_. After 24 h, the RHE tissues were topically exposed to the samples at 16 ± 0.5 µL/well for 42 min at room temperature. The tissues were then subsequently rinsed 25 times using sterile DPBS. Then, they were incubated for 42 h at 37 ± 0.5 °C under 5% CO_2_ in 2 mL of growth medium for the MTT reduction experiment, as described in Section 2.9.3.

#### 2.9.3. Viability of Skin Fibroblast Cells and SkinEthic RHE Tissue

Cytotoxicity was determined by measuring the dehydrogenase activity of viable keratinocytes following a 42 h post-incubation. The activity was determined after incorporation of MTT, as described previously [55]. Each cell line and RHE tissue was treated with 1 mg/mL MTT solution for 3 h. The blue formazan crystals were subsequently extracted from the cells and tissues using isopropanol for at least 2 h. The concentration of formazan was measured by determining the optical density (OD) at 570 nm using a spectrophotometer (FlexStation 3, Molecular Devices, California, CA, USA).

Each experiment was performed in triplicate. After subtracting the blank OD from all raw data, the mean OD values ± SDs were calculated using three measurements per test substance, and the percentage of cell viability was expressed using Equation (6), relative to that for the NC. The NC value was set at 100%.

(6) $$\mathrm{Viability}\;\left(\%\right)\;=\;\frac{{\mathrm{Mean}\;\mathrm{OD}}_{\mathrm{treated}}}{{\mathrm{Mean}\;\mathrm{OD}}_{\mathrm{control}}}\;\times \;100$$

#### 2.9.4. Morphological Evaluation

Morphological evaluation was performed to compare the morphologies of the native and nanoparticle-treated two-fibroblasts during irritation studies. To obtain morphologies of nanoparticle-treated fibroblast, after the process of sample exposure for 15 min as described in Section 2.9.1, the fibroblasts were observed under a Zeiss Axiovert 40 C microscope (Carl Zeiss, Göttingen, Germany) with a 100× objective.

### 2.10. In Vitro Permeation and Retention Studies Using SkinEthic RHE Tissue

#### 2.10.1. Tissue Preparation

Upon receipt of the SkinEthic RHE tissues, which had been grown in a 24-well plate format, culture inserts were removed from the nutrient gel and transferred under aseptic conditions into a new sterile 24-well plate containing 1.5 mL of a maintenance medium provided by the manufacturer. The tissues were incubated at 37 ± 0.5 °C in a humidified atmosphere with 5% CO_2_. After 48 h, the culture inserts were placed into a new sterile 6-well plate containing 3 mL of PBS (pH 7.4, receptor medium), following which the permeation tests were conducted.

#### 2.10.2. In Vitro Permeation Studies

Permeation tests were performed using SkinEthic RHE tissues in 6-well plates containing 3 mL of the receptor medium. We placed 0.3 mL of each sample on the epidermal surface and then placed the well plates containing tissues in an incubator at 37 ± 0.5 °C under a humidified atmosphere with 5% CO_2_ and shaken at 100 rpm (NB-101SRC, N-BIOTEK, Gyeonggi-do, Korea). At predetermined time intervals (0.5, 1, 2, 4, and 8 h), aliquots of 1 mL were withdrawn from the basolateral side in the well of the plate, passed through 0.45-μm membrane filters, and immediately analyzed using HPLC, as described in Section 2.8.

#### 2.10.3. In vitro Skin Tissue Retention Studies

Following in vitro permeation study, the amount of AD retained in the SkinEthic RHE tissues was investigated using HPLC. Following the skin permeation experiments, the tissues were transferred to a glass vial (5 mL) and mixed with 2 mL of maintenance media. Then the tissue suspension was homogenized using high speed homogenizer at 10,000 rpm. Aliquots of 1 mL were withdrawn from the vial, passed through 0.45-μm PTFE membrane filters, and immediately analyzed for AD using HPLC, as described in Section 2.8.

### 2.11. Statistical Analysis

All experiments were performed in triplicates. The presented data (means ± SDs) were compared by one-way analysis of variance and Student’s t-tests. A value of *p* < 0.05 was considered statistically significant.

## 3. Results and Discussion

### 3.1. Characterization of AD-Loaded SLNs

#### 3.1.1. Nanoparticle Size, PDI, and Zeta Potential

**Figure 1:**
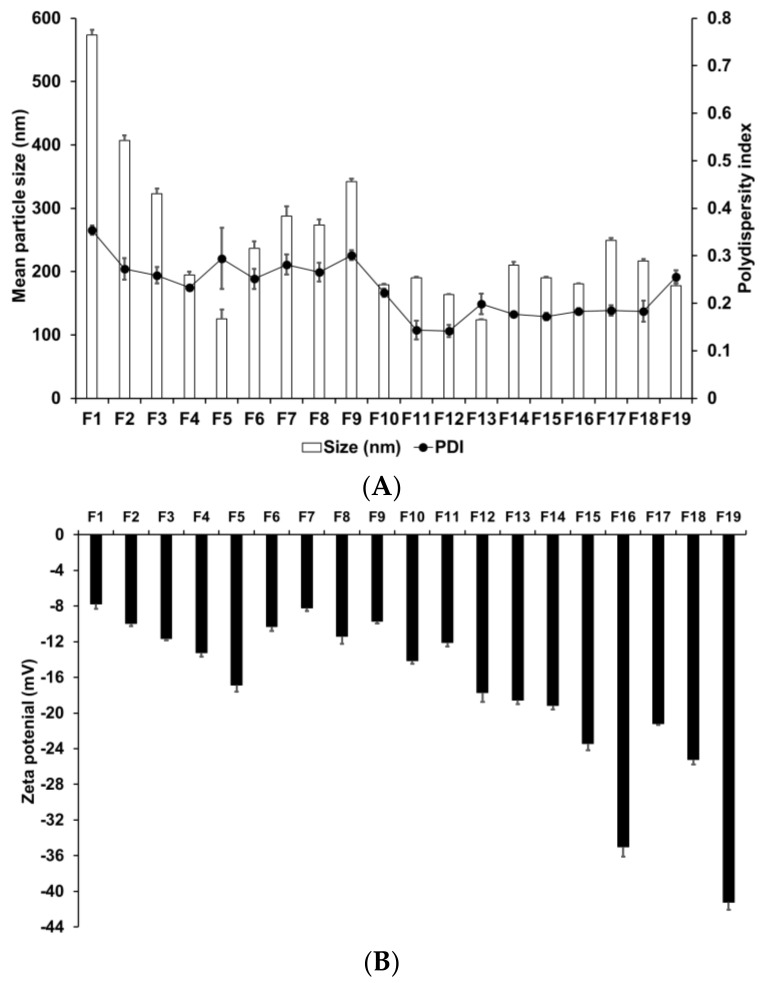
Physicochemical characteristics (average particle size, polydispersity index (PDI), and zeta potential) of adenosine-loaded solid lipid nanoparticles (SLNs). Results are expressed as the means ± standard deviations of three independent experiments (*n* = 3). (**A**) Particle size and PDI and (**B**) zeta potential of adenosine-loaded SLNs prepared using different materials and compositions of adenosine, lipids, and surfactants. PDI, polydispersity index; SLNs, solid lipid nanoparticles.

We evaluated the effects of various materials and AD, lipid, and surfactant compositions on the formation of nanoparticles. Zeta potential is an important parameter that provides information regarding the storage stability of nanoparticles [62]. High zeta potential ensures high energy barrier and favors high storage stability, whereas low zeta potential implies easy release the encapsulated drugs [63]. Therefore, a moderated zeta potential plays an important role in a balance between storage stability and drug release. Figure 1 presents the particle size, PDI, and zeta potential of SLNs F1 to F5, which were prepared using different solid lipids. Among these lipids, those with a longer carbon chain tended to produce smaller and more homogeneous particles; additionally, these particles were more stable, as indicated by their higher zeta potential. This suggests that lipids with longer carbon chains present a greater inherent negative charge from the carboxyl end group of saturated fatty acids [64]. The higher negative charge on the particle surface leads to increased repulsion among the particles; therefore, the tendency for aggregation is lower in these particles than in those with a lower negative charge.

The particle size, PDI, and zeta potential of F4, F6, F7, F8, and F9, which were formed using different surfactants, demonstrated that F4 had the smallest particle size and the most homogeneous and stable formulation. Lower particle size and higher zeta potential were obtained by either decreasing the hydrophilic–lipophilic balance (HLB) of the lipophilic surfactant or increasing the HLB of the hydrophilic surfactant. In addition, hydrophilic surfactants induced a higher influence on particle characteristics (size, PDI, and zeta potential) than lipophilic surfactants. This suggests that hydrophilic surfactants stabilize the interface between W_1_/O and W_2_, whereas lipophilic surfactants stabilize the interface between W_1_ and O; therefore, the effect of the hydrophilic surfactant on particle characteristics was stronger.

We evaluated the effect of drug, lipid, and lipophilic surfactant concentrations on the particle size of F4 and F10 to F19. An increase in the concentration of AD or SA increased the particle size. For lipophilic surfactants, an increase in the concentration of SP 80 decreased the particle size. The PDI was low for all aforementioned formulations. The use of different concentrations of SA and SP 80 did not produce any significant effect on the PDI. The zeta potential increased with an increase in the concentration of SA or SP 80. This suggests that the effect of lipid composition on the particle size could be related to the interaction between the selected lipid and surfactant [65]. The zeta potential values indicated that a higher amount of SA or SP 80 tended to improve the stability of SLNs.

**Figure 2:**
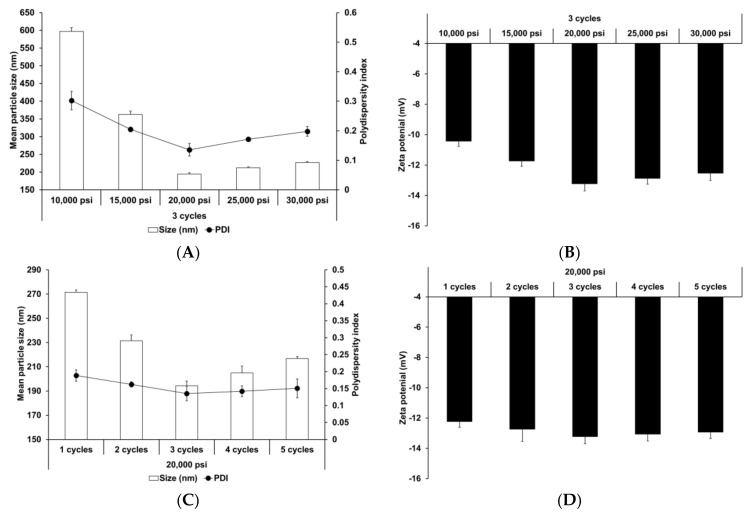
Physicochemical characteristics (average particle size, polydispersity index (PDI), and zeta potential) of adenosine-loaded solid lipid nanoparticles (SLNs). Results are expressed as the means ± standard deviations of three independent experiments (*n* = 3). (**A**) Particle size and PDI and (**B**) zeta potential of adenosine-loaded SLNs prepared using different pressures. (**C**) Particle size and PDI and (**D**) zeta potential of adenosine-loaded SLNs prepared using different number of cycles. PDI, polydispersity index; SLNs, solid lipid nanoparticles.

The effect of HPH parameters (homogenization pressure and number of homogenization cycles) was also evaluated. HPH has been widely used to prepare nanoparticles and nano-emulsions [66,67]. Reduction of particle size improves the shelf life of such products by reducing aggregation and creaming rate [68]. Figure 2 presents the particle size, PDI, and zeta potential of SLNs prepared using different HPH parameters. Upon increasing the homogenization pressure and number of homogenization cycles to 20,000 psi and 3, respectively, the particle size and PDI of SLNs decreased, followed by a slight increase, while the zeta potential increased, followed by a slight decrease. This suggests that although the HPH process contributed to particle size reduction and improvement in particle stability, over-processes, such as excessive pressure and cycles, lead to reduction in process efficiency. Particle size and size distribution were significantly affected by HPH pressure. Therefore, excessive pressure may lead to droplets cracking, as reported in other studies [69,70]. In case of excessive cycles, increasing number of homogenization cycles lead to formation of rapidly aggregating droplets during HPH [71,72].

#### 3.1.2. Determination of Drug-Loading Capacity

**Figure 3:**
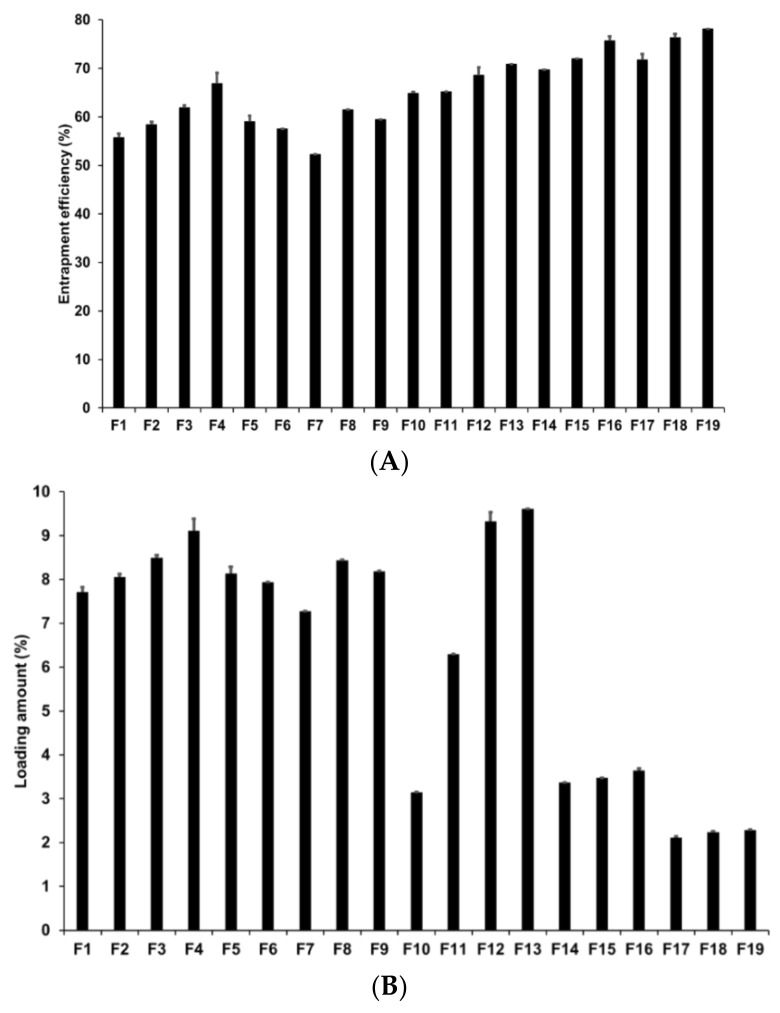
Loading efficiency and loading amount of AD-loaded SLNs. Results are expressed as the means ± standard deviations of three independent experiments (*n* = 3). (**A**) Loading efficiency and (**B**) loading amount of AD-loaded SLNs prepared using different compositions of AD, lipids, surfactants. AD, adenosine; PDI, polydispersity index; SLNs, solid lipid nanoparticles.

Loading capacity (LE and LA) is an important parameter in the formulation of lipid nanoparticles as it affects drug diffusion in the lipid matrix and drug retention in the skin [73]. In addition, a high loading capacity might be beneficial for reducing skin irritation caused by the drug by avoiding direct contact between the drug and skin surface [74]. In our study, loading capacity was evaluated by determining the LE and LA of AD-loaded SLNs using the centrifugation method followed by concentration estimation using the HPLC method. Figure 3 presents the LE and LA of SLNs prepared by different solid lipids. Our results for F1 to F5 revealed that F4 had the highest LE and LA at 66.86% and 9.11%, respectively. The LE and LA of F3 were the second highest at 61.87% and 8.49%, respectively. This suggests that longer chain fatty acids entrap a greater amount of AD despite the fact that the log *p* value of AD is −1.05. The reason for this might be because of the second emulsification process to form W_1_/O/W_2_ phase with high-speed homogenization and to control particle sizes with high-pressure homogenization. In this process, the drug in-water phase might be lost but we speculate that the lipids with longer fatty acid chains efficiently prevented the loss of the drug because the liberation of the hydrophilic drug might be more difficult within the lipids with longer fatty acid chains than within those with shorter fatty acid chains.

Among F4, F6, F7, F8, and F9, which were prepared using different surfactants, F4 exhibited the highest loading capacity. Higher loading capacity was attributed to either lower HLB of the lipophilic surfactants or higher HLB of the hydrophilic surfactants. Additionally, lipophilic surfactants induced greater influence on the loading capacity than hydrophilic surfactants. This suggested that lipophilic surfactants stabilized the interface between W_1_ and O and, therefore, a higher amount of AD was stably dispersed in the lipid matrix when preparing the primary W_1_/O emulsion.

Considering the effects of the concentrations of the drug, lipid, and lipophilic surfactant, the overall percentages of LE and LA of F4 and F10 to F19 were 64.85–78.12% and 2.11–9.61%, respectively. An increase in drug, lipid, and surfactant concentrations increased the LE of AD in SLNs. However, although the LA of AD increased with an increase in the drug and surfactant concentrations, it decreased with an increase in the lipid concentration. According to HLB scale, primary W_1_/O emulsions prepared at SA-to-SP 80 ratios of 1:0.04, 1:0.2, and 1:1 exhibited HLB values of 5.93, 5.72, and 5.15 respectively. Among the W_1_/O/W_2_ emulsions, F14 to F19 had HLB values ranging from 9.13 to 14.94, which are part of O/W emulsifier, while that of the formulations F4, F12, and F13 ranged from 17.08 to 21.11, which are part of solubilizer or hydrotrope [75]. When preparing the primary W_1_/O and W_1_/O/W_2_ emulsions, the emulsion with higher lipophilicity exhibited higher LE and LA. However, the LA decreased with an increase in the SA concentration. Therefore, although AD is a hydrophilic drug (log P = −1.05), an emulsion with high lipophilicity can stably encapsulate hydrophilic drugs into lipophilic materials [76,77].

**Figure 4:**
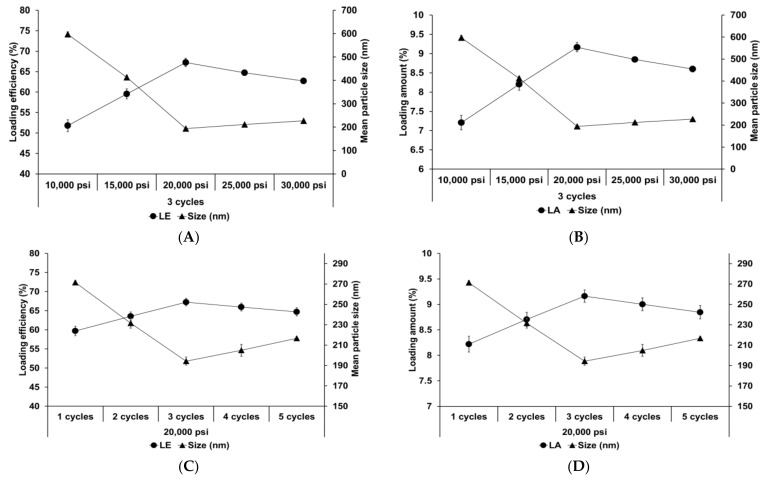
Loading capacity of AD-loaded SLNs prepared using different parameters for homogenization. Results are expressed as the means ± standard deviations of three independent experiments (*n* = 3). (**A**) Loading efficiency (LE) and particle size and (**B**) LA and particle size of AD-loaded SLNs prepared using different pressures. (**C**) LE and particle size and (**D**) loading amount (LA) and particle size of AD-loaded SLNs prepared using different number of cycles. AD, adenosine; SLNs, solid lipid nanoparticles; LE, loading efficiency; LA, loading amount.

Figure 4 shows the effect of different homogenization pressures and number of homogenization cycles on SLNs. Both LE and LA increased with an increase in both the homogenization pressure and number of homogenization cycles to 20,000 psi and 3 cycles, respectively, followed by a slight decrease. The particle size showed an inverse relationship with the loading capacity. This suggests that the reduction in particle size induces an increase in the number of particles. Therefore, although the drug-loading capacity of a lipid particle decreases due to reduction of the lipid matrix, the drug-loading capacity for the entire SLN increases. Considering the effects of over processes, the induction of droplet cracking [69,70] and rapid droplet aggregation [71,72] leads to a decrease in the loading capacity. Indeed, Yang et al. reported similar results that the loading capacity of emulsions increased with an increase in the homogenization pressure and number of homogenization cycles up to 100 MPa and 4 cycles, respectively, and subsequently decreased with further increase in these parameters [71].

### 3.2. Mechanical Properties of Elastic Artificial Skin

#### 3.2.1. Viscosity Behaviors of Synthesized Elastic Artificial Skin

**Figure 5:**
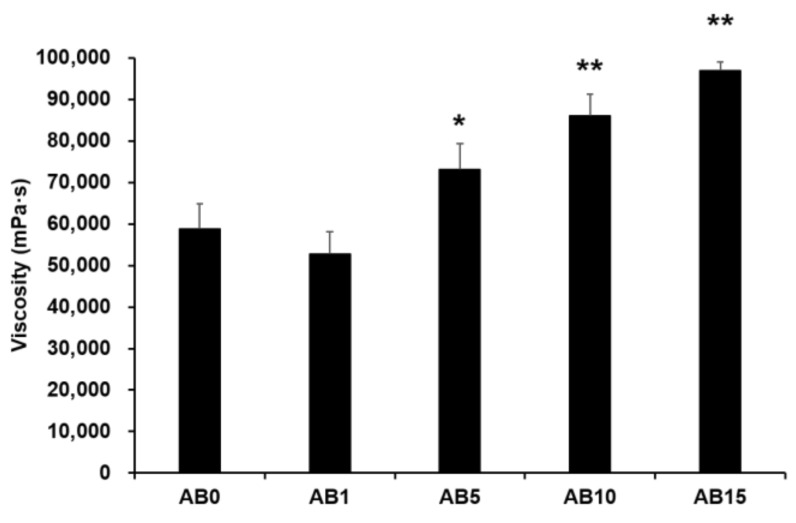
Viscosity of the elastic artificial skin. Results are expressed as the means ± standard deviations of three independent experiments (*n* = 3). AB0: Non-SLN artificial skin; AB1: Artificial skin with 1% SLNs; AB5: Artificial skin with 5% SLNs; AB10: The artificial skin with 10% SLNs; AB15: Artificial skin with 15% SLNs. Statistical significance of the difference in viscosity between the formulation without SLNs, and formulations with SLNs is indicated by either a single asterisk (*p* < 0.05) or double asterisks (*p* < 0.01). SLNs, solid lipid nanoparticles.

Viscosity was measured to evaluate the possible effect of variations in the formulation of elastic artificial skin with/without AD-loaded SLNs. Spreadability plays an important role in topical formulations. When applying elastic artificial skin to the desired skin area, good spreadability is desirable [78]. However, to reduce the cross-link reaction time between components A and B when synthesizing elastic artificial skin, an increase in the viscosity profiles of formulations is beneficial. The viscosity profiles of elastic artificial skin were evaluated using shear experiments. Figure 5 shows results regarding the viscosity of elastic artificial skin with/without AD-loaded SLNs. An increase in the proportion of SLNs in the elastic artificial skin significantly increased the viscosity. The viscosity value of the formulation containing SLN 1% was lower compared to that without SLN but the difference was not statistically significant. Reportedly, an increase in the composition of SLN gradually increased the viscosity of the elastic artificial skin due to an increase in the oil phase in SLNs [77]. In addition, among the evaluated elastic artificial skin formulations, AB5, AB10, and AB15 showed a significant increase in viscosity at *p* < 0.05, *p* < 0.01, and *p* < 0.01, respectively.

#### 3.2.2. Thickness of Elastic Artificial Skin

**Figure 6:**
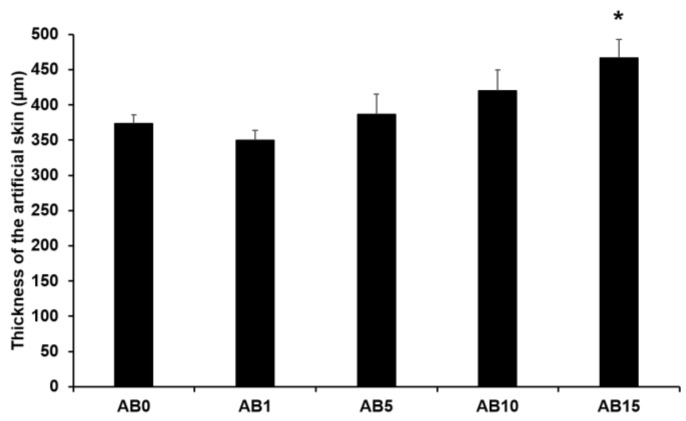
Thickness of the elastic artificial skin. Results are expressed as the means ± standard deviations of three independent experiments (*n* = 3). AB0: Non-SLN artificial skin; AB1: Artificial skin with 1% SLNs; AB5: Artificial skin with 5% SLNs; AB10: Artificial skin with 10% SLNs; AB15: Artificial skin with 15% SLNs. Statistical significance of the difference in thickness between the elastic artificial skin without SLNs and the formulations with SLNs was indicated by a single asterisk (*p* < 0.05).

Figure 6 presents the thickness of the elastic artificial skin with/without AD-loaded SLNs at different concentrations. The thickness tended to gradually increase with increasing SLN concentration exceeding 5%. The main reason for this might be that the increased concentration of SLN in the elastic artificial skin increased the viscosities. A higher viscosity of the elastic artificial skin compositions could demonstrate stronger resistance against the spread forces applied to the compositions when the artificial skins were synthesized. Therefore, the thickness of the elastic artificial skin was increased when the viscosity of the formulations was increased [79,80]. The difference in thickness of AB0 and of AB15 exhibited a statistical significance of *p* < 0.05.

#### 3.2.3. Tensile Strength (TS), Elongation at Break (EAB), and Young’s Moulus (YM)

The mechanical properties (TS, EAB, and YM) of the elastic artificial skin with/without AD-loaded SLNs are presented in Table 6. We found that the addition of 1% SLNs increased the TS of the elastic artificial skin, while an increase in the concentration of SLNs decreased the TS. EAB is usually related to the cross-linked network of the elastic artificial akin and the intermolecular force [81]. An increase in the concentration of SLNs increased the EAB of the elastic artificial skin. This suggests that the effect of viscosity was stronger than that of the cross-linked network. Generally, increased TS values are followed by a decrease in EAB values [82]. Moreover, we noted that an increase in the concentration of SLN decreased the YM of the elastic artificial skin. This suggests that the effect of TS was stronger than that of EAB.

### 3.3. In Vitro AD-Release Studies

**Figure 7:**
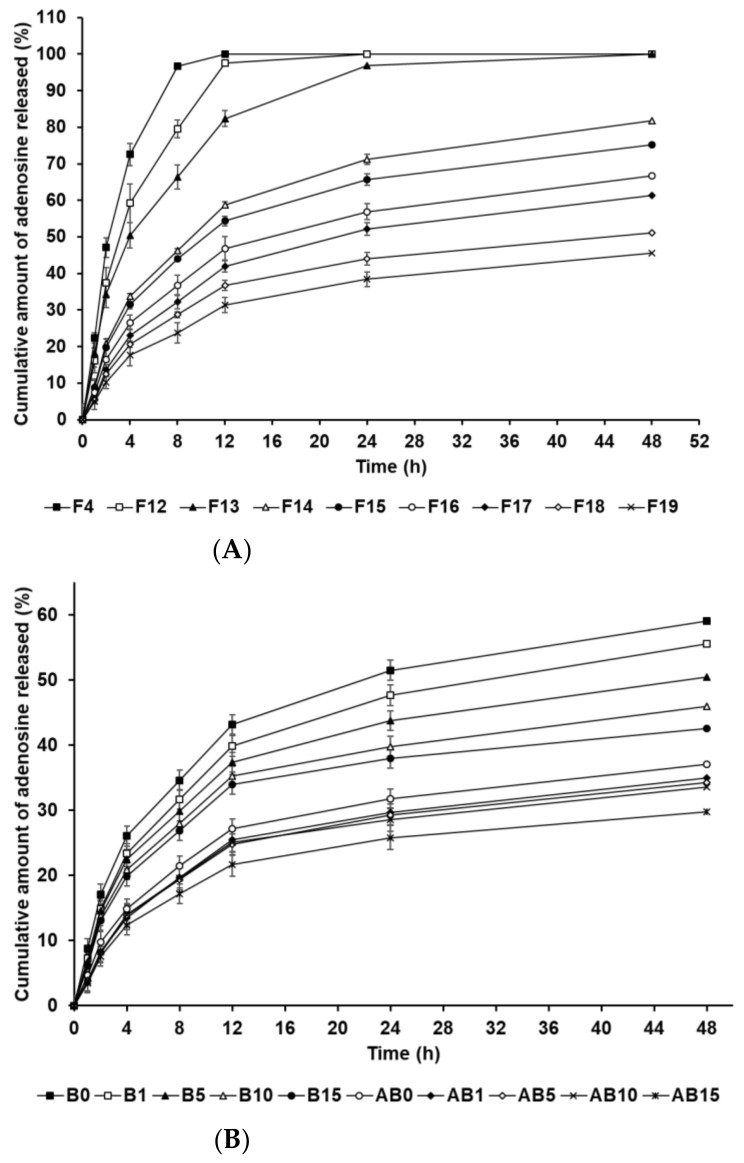
Cumulative percentage release profiles of adenosine from (**A**) solid lipid nanoparticles and (**B**) the synthesized elastic artificial skin with/without SLNs in release medium, as determined using dialysis bag method. Results are expressed as the means ± standard errors of three independent experiments (*n* = 3). B0: non-SLN B formulation; B1: B formulation with 1% SLNs; B5: B formulation with 5% SLNs; B10: B formulation with 10% SLNs; B15: B formulation with 15% SLNs; AB0: Non-SLN artificial skin; AB1: Artificial skin with 1% SLNs; AB5: Artificial skin with 5% SLNs; AB10: Artificial skin with 10% SLNs; AB15: The artificial skin with 15% SLNs.

The in vitro release profile of AD-loaded SLNs and the elastic artificial skins was determined using the dialysis membrane method. Figure 7A shows the drug release from F4 and F12 to F19. F4, F12, and F13, which contained 0.5 g of SA, demonstrated burst release. F14 to F19, which contained 1.5 g and 2.5 g of SA, showed burst release over 12 h and then sustained release until 48 h. Considering the effect of the oil-phase concentration in the SLNs, an increase in SA and SP 80 concentrations increasingly resulted in sustained release. This suggests that increased concentrations of SA and SP 80 can enable higher drug loading into the particle core; therefore, the enriched amount of AD in the particle core may prolong the release rate [83,84]. In addition, the lipophilic surfactant might act as an obstacle of drug diffusion, which resulted in the slower drug release [85,86]. The biphasic release profiles of the formulations F14 to F19, which contained 1.5 g and 2.5 g of SA, might be attributed to the type of AD loaded into the SLN. The first release, which was burst, was attributed to AD on the surface of lipid nanoparticles that enabled rapid release. Following initial burst release, the release of AD encapsulated in the inner core of SLNs was delayed and occurred in a sustained manner. This suggests that an increase in the lipophilicity of the formulation, as indicated by HLB values, leads to delayed drug release because the amount of AD loaded in the particle core is higher than that on the particle surface [87,88].

Figure 7B presents the in vitro release profile curves of AD in the elastic artificial skins synthesized via the reaction between components A and B (AB0, AB1, AB5, AB10, and AB15) and component B (B0, B1, B5, B10, and B15). The AD-release profile for the synthesized elastic artificial skin and B component with/without AD-loaded SLNs demonstrated sustained release for 48 h. Concretely, the AD-release profiles of synthesized elastic artificial skins and component B showed burst release for 12 h, followed by sustained release until 48 h. The amount of AD released was lower for the synthesized elastic artificial skin than that for the component B, that is, the AD release was faster for component B. After 48 h, the highest release of AD was exhibited by B0 at 59.10%, followed by B1 at 55.57%. For the synthesized elastic artificial skin, the highest concentration of AD released was from AB0 at 37.13%, followed by that from AB1 at 34.94%. The delayed release with an increase in SLN concentration might be attributed to the increase in viscosity of the elastic artificial skin. This suggests that the high viscosity of the formulation decreased drug diffusion. Therefore, the anti-wrinkle effect could be prolonged. When comparing the synthesized elastic artificial skin with the B component, the release rate of AD from the synthesized elastic artificial skin was lower than that from component B. This result might be attributed to the formed membrane provided by the elastic artificial skin. The lag in AD release is attributed to the reaction between component A and B because the migration of AD in the cross-linked elastic artificial skin was more difficult than that in component B alone [89]. In a drug-release study by Prokopowicz et al., the higher viscosity of polydimethylsiloxane film led to a decrease in drug-release rate [90]. Consequently, the release of AD in the elastic artificial skin was delayed.

### 3.4. In Vitro Irritation Studies

#### 3.4.1. Irritation Study and Morphological Evaluation Using Skin Fibroblast Cell Lines

**Figure 8:**
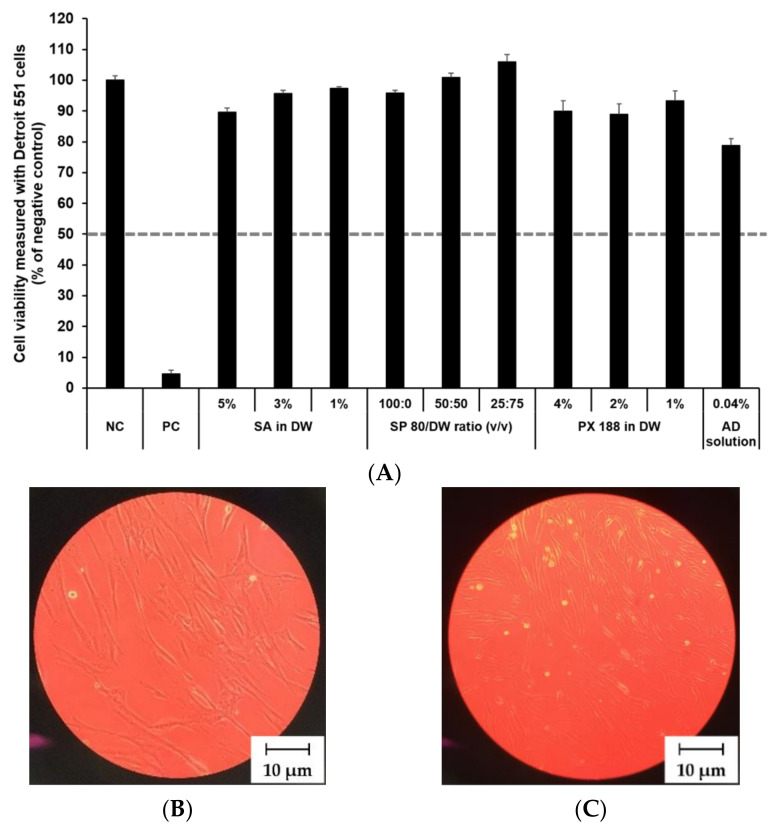
Irritation study and morphological evaluation using Detroit 551 cell line. (**A**) Viability of Detroit 551 cells treated with SLN components and AD solution, (**B**) the native morphology of Detroit 551 cells, and (**C**) nanoparticle-treated morphology of Detroit 551 cells. Cell viability was measured using MTT assay. The values greater than 50% (dotted line) indicate that test materials are non-irritant to the skin. Results are expressed as mean ± standard deviation of three independent experiments (*n* = 3). NC: DPBS; PC: 5% SDS solution; SA: Stearic acid; DW: Distilled water; SP 80: Span 80; PX 188: Poloxamer 188, AD: Adenosine.

**Figure 9:**
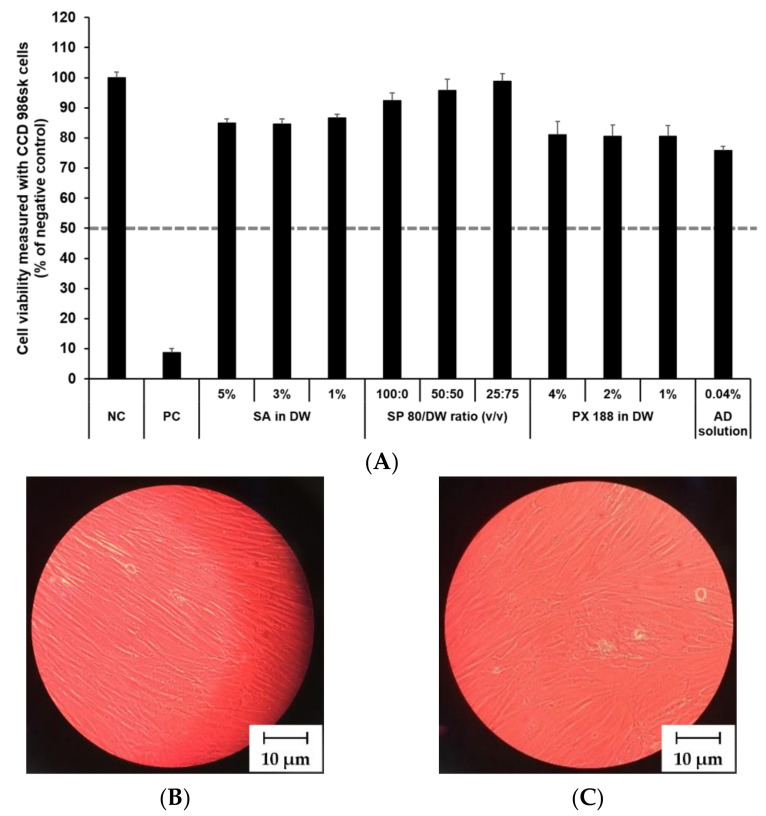
Irritation study and morphological evaluation using CCD 986sk cell line. (**A**) Viability of CCD 986sk cells treated with SLN components and AD solution, (**B**) the native morphology of CCD 986sk cells, and (**C**) nanoparticle-treated morphology of CCD 986sk cells. Cell viability was measured using MTT assay. The values greater than 50% (dotted line) indicate that test materials are non-irritant to the skin. Results are expressed as the means ± standard deviations of three independent experiments (*n* = 3). NC: DPBS; PC: 5% SDS solution; SA: Stearic acid; DW: Distilled water; SP 80: Span 80; PX 188: Poloxamer 188; AD: Adenosine.

The cytotoxicity of SLN components and AD at various concentrations was investigated by MTT assay as shown in Figure 8 and Figure 9. The prediction model for skin irritants was based on the European classification system (ECS). According to ECS, all test substances were demonstrated to be non-skin irritants for the fibroblast cell lines CCD 986sk and Detroit 551, because the mean cell viability for each test substance was over 50% (Figure 8A and Figure 9A). Microscopy was used to compare the morphology of native and nanoparticle-treated fibroblast. Figure 8B and Figure 9B shows the native fibroblasts, respectively, while Figure 8C and Figure 9C shows the nanoparticle-treated fibroblasts, respectively. The morphology of nanoparticle-treated fibroblasts was almost the same as the initial morphology.

In this study, the mean OD values for DPBS, the NC, were 1.37 ± 1.11 (CCD 986sk) and 1.69 ± 0.11 (Detroit 551), meeting the acceptance criteria of OD > 1.2. The mean viability values for 5% SDS solution, the PC, were 8.71 ± 0.46% (CCD 986sk) and 4.64 ± 0.14% (Detroit 551), which satisfied the acceptance criteria of viability < 40%.

#### 3.4.2. Irritation Study Using SkinEthic RHE Tissue Model

**Figure 10:**
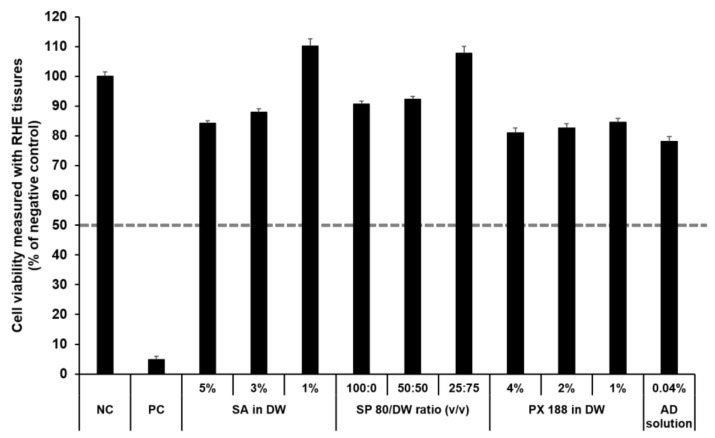
Viability of SkinEthic RHE tissue treated with SLN components and AD solution. The cell viability was measured using MTT assay. The values greater than 50% (dotted line) indicate that test materials are non-irritant to the skin. Results are expressed as means ± standard deviations of three independent experiments (*n* = 3). NC: DPBS; PC: 5% SDS solution; SA: Stearic acid; DW: Distilled water; SP 80: Span 80; PX 188: Poloxamer 188; AD: Adenosine.

**Figure 11:**
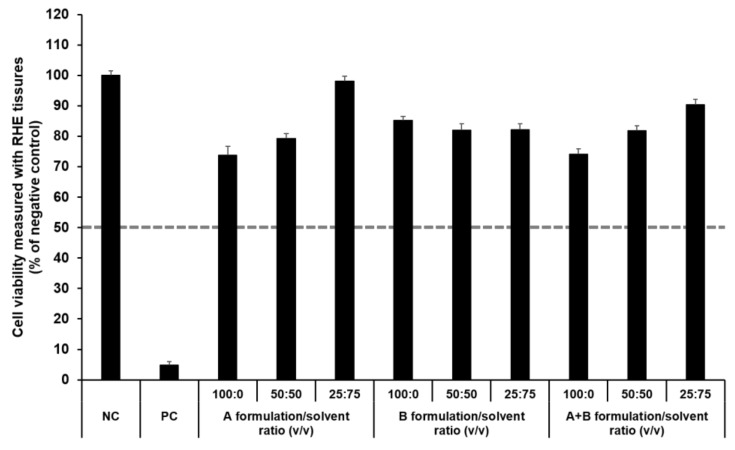
Viability of SkinEthic RHE tissue treated with components of the elastic artificial skin. The cell viability was measured using MTT assay. The values greater than 50% (dotted line) indicate that test materials are non-irritant to the skin. Results are expressed as means ± standard deviations of three independent experiments (*n* = 3).

The SkinEthic RHE, a reconstructed human skin model, is being evaluated in the ECVAM Skin Irritation Validation Study [55]. The MTT assay was used to measure cell viability in the epidermis and was expressed as the percentage normalized to the viability with the NC (DPBS), which was set at 100%. According to the EU classification, if the percentage viability is >50%, the substance is classified as non-irritant. However, if the percentage viability is ≤50%, the substance is classified as an irritant. All test substances were non-skin-irritating substances, as shown in Figure 10 and Figure 11, as the mean tissue viability for each test substance was greater than 50%.

In accordance with the SkinEthic Skin Irritation Test SOP, results of MTT assay for the NC (DPBS) and PC (5% SDS) should meet the acceptance criteria. The mean absorbance value of NC should be ≥1.2 at 570 nm. For PC, the mean viability expressed as percentage of the NC should be <40%. In this study, the mean absorbance value of NC was 1.57 ± 0.01, and the mean viability following treatment with PC was 4.84 ± 0.09%; both values met the acceptance criteria, as per the SkinEthic Skin Irritation Test SOP.

### 3.5. In Vitro Permeation Studies Using SkinEthic RHE Tissue

#### 3.5.1. In Vitro Permeation Studies

**Figure 12:**
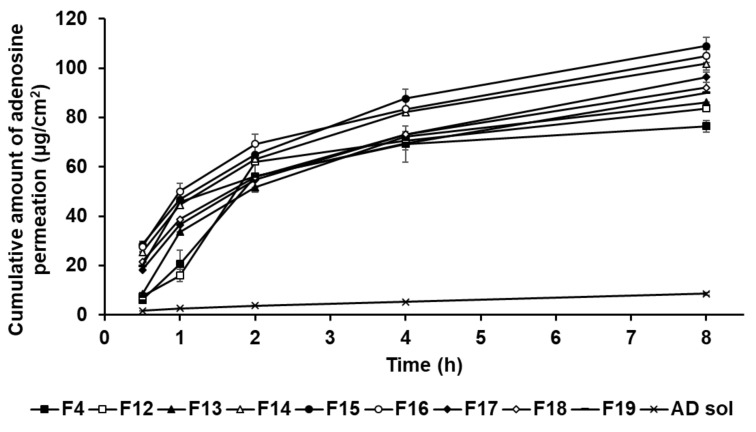
Cumulative permeation profiles for adenosine from solid lipid nanoparticles into the medium. Results are expressed as the means ± standard deviations of three independent experiments (*n* = 3).

In this study, the formulations of AD-loaded SLNs, the elastic artificial skin with/without SLNs, and AD solution compared those AD permeation effects. The SkinEthic RHE model was cultured as a 0.5 cm^2^ insert. Figure 12 presents the permeation profiles of AD incorporated in SLNs F4 and F12 to F19 for 8 h. All SLNs could cause greater permeation of AD across the SkinEthic RHE tissue compared to AD solution. The cumulative amount of AD permeation of F15 was the highest among the tested SLNs and was approximately 13 times higher than that for the AD solution. The cumulative amount of AD permeation of F16 was the second highest at approximately 12 times that of AD solution. The order of permeation amount of AD after 8 h was F15 > F16 > F14 > F17 > F18 > F19 > F13 > F12 > F4 > AD solution. Considering the surfactants in SLN, an increase in the concentration of SP 80 tended to increase the cumulative amount of AD permeation when the concentration of SA was increased up to 1.5 g, that is, for F4 and F12 to F15. However, AD permeation from the F16 to F19 tended to decrease with increasing SP 80 concentration. This suggests that an increase in the amount of oil phase increased the viscosity of the formulation and, therefore, the permeation of AD from SLNs was delayed [83]. Regarding the lipids in SLNs, the cumulative amount of AD permeation into the SkinEthic RHE tissue increased when the concentration of SA was increased up to 1.5 g, that is, for F4 and F16; however, the cumulative amount of AD permeation decreased for the formulations containing 2.5 g of SA, that is, F17 to F19. This suggests that SLNs with higher lipophilicity improved the permeation of AD into the epidermis owing to the affinity between SLNs and intercellular lipids in stratum corneum [91]. However, the SLNs comprising 2.5 g of SA, as a high amount of lipids, were the high viscosity and therefore delayed drug permeation into the epidermis [31,83]. Therefore, although SA can enhance the permeation of active ingredients, optimization of the SA-to-SP 80 ratio is crucial for developing SA-based SLNs with high permeation into the epidermis.

**Figure 13:**
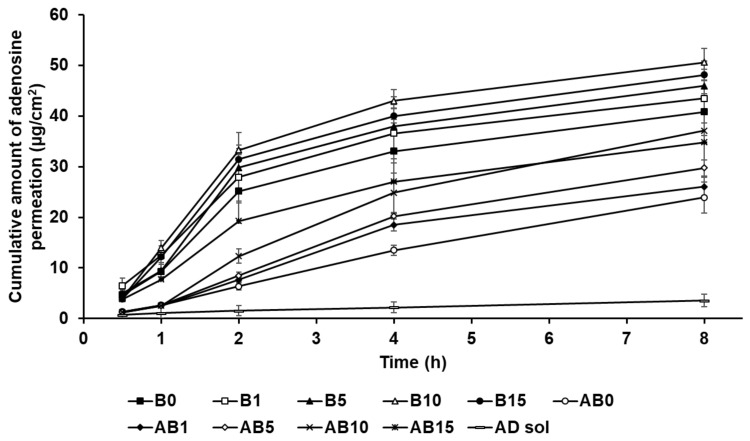
Cumulative permeation profiles of AD from the synthesized elastic artificial skin with/without SLNs in the medium. Results are expressed as the means ± standard errors of three independent experiments (*n* = 3). B0: non-SLN B formulation; B1: B formulation with 1% SLNs; B5: B formulation with 5% SLNs; B10: B formulation with 10% SLNs; B15: B formulation with 15% SLNs; AB0: Non-SLN artificial skin; AB1: The artificial skin with 1% SLNs; AB5: The artificial skin with 5% SLNs; AB10: The artificial skin with 10% SLNs; AB15: The artificial skin with 15% SLNs.

Figure 13 shows the permeation of AD from component B (B0, B1, B5, B10, and B15) and the synthesized elastic artificial skin formed through the reaction between components A and B (AB0, AB1, AB5, AB10, and AB15) in the SkinEthic RHE model. The cumulative amount of AD permeation for all tested formulations of the elastic artificial skin over 8 h was higher than that for AD solution. The cumulative amount of AD permeation from the elastic artificial skins with SLNs into the SkinEthic RHE tissue were higher than those for formulations without SLNs. The order of permeation of AD after 8 h was B10 > B15 > B5 > B1 > B0 > AB10 > AB15 > AB5 > AB1 > AB0 > AD solution. This suggests that nano-sized SLNs provided a larger interfacial surface area, enabling close contact between SLNs and the skin, thus facilitating AD permeation into the skin [77,92]. In addition, the lipid property of SLNs enabled effective permeation of AD into the skin via intercellular lipid route through the epidermis and then exhibited sustained drug release [83,91]. Therefore, the elastic artificial skins using SLNs effectively delivered AD into the skin. Increasing SLN concentration in the elastic artificial skin tended to increase AD permeation. After 8 h, B10 exhibited the greatest amount of AD permeation at 50.59 µg/cm^2^, followed by B15 at 48.13 µg/cm^2^; these values were approximately 6 and 5.5 times higher, respectively, than those of AD solution. In case of the synthesized elastic artificial skin, AB10 exhibited the highest amount of AD permeation at 37.14 µg/cm^2^, followed by AB 15 at 34.79 µg/cm^2^; the values were approximately 5 and 4 times higher, respectively, than those for the AD solution. This suggests that the high amount of lipid increased the viscosity and, thereby, limited the independent migration of SLNs [31]. Therefore, the formulations B15 and AB15 exhibited delayed permeation profiles in the epidermis than B10 and AB10. The amount of AD permeated from the synthesized elastic artificial skin was lower than that from B component formulations. This result might be attributed to the membrane effect provided by the elastic artificial skin [89,90].

#### 3.5.2. Skin Tissue Retention Studies

**Figure 14:**
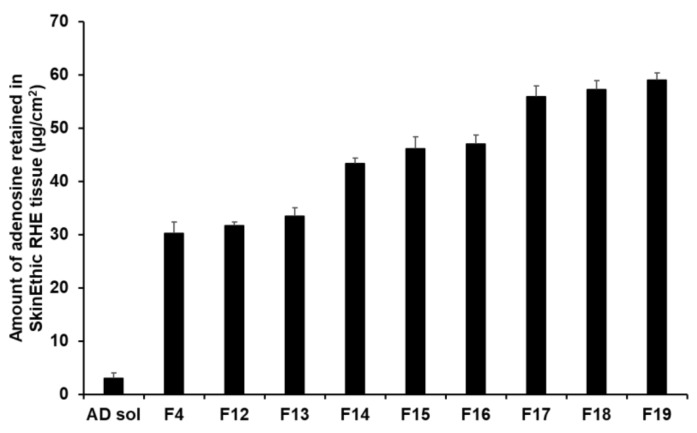
Results for AD retention from AD-loaded SLNs in SkinEthic RHE tissue after permeation studies. Results are expressed as means ± standard deviation of three independent experiments (*n* = 3).

The retention results demonstrated that an increase in the composition of SA and SP 80 tended to increase the amount of AD retained (Figure 14). In this sense, F17 to F19 ensured a higher amount of AD in the tissues, ranging from 55.89 µg/cm^2^ (23.29%) to 59.06 µg/cm^2^ (24.61%). This suggests that owing to the lipid property of SLNs, they physically interacted with intercellular lipids in the stratum corneum and then effectively created a drug reservoir [77,93]. Owing to this reservoir, the drug was consistently released. Because lipids can form a thin lipid membrane on the skin, use of large lipid amount could not improve permeation of the active ingredient; however, this approach could achieve sustained release of the active ingredient and prevent water loss from the skin [91,93].

**Figure 15:**
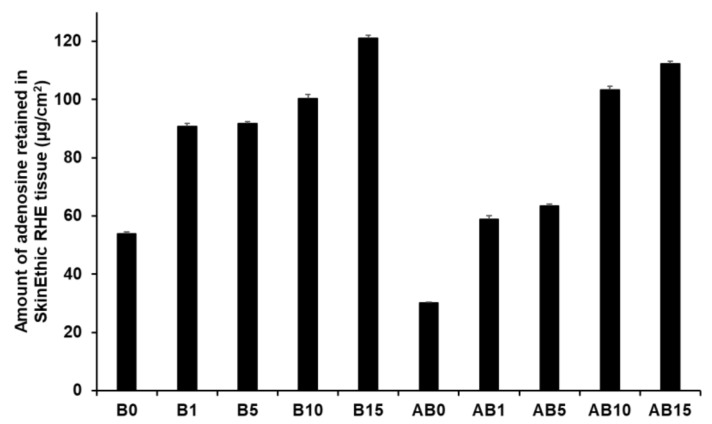
Retention studies for the synthesized elastic artificial skin with/without SLNs in SkinEthic RHE model after permeation studies. Results are expressed as means ± standard deviations of three independent experiments (*n* = 3). B0: Non-SLN B formulation; B1: B formulation with 1% SLNs; B5: B formulation with 5% SLNs; B10: B formulation with 10% SLNs; B15: B formulation with 15% SLNs; AB0: Non-SLN artificial skin; AB1: Artificial skin with 1% SLNs; AB5: Artificial skin with 5% SLNs; AB10: Artificial skin with 10% SLNs; AB 15: Artificial skin with 15% SLNs.

Figure 15 presents the amount of AD retained in the skin for both component B (B0, B1, B5, B10, and B15) and synthesized elastic artificial skins formed by reaction between components A and B (AB0, AB1, AB5, AB10, and AB15). An increase in SLN concentration in the elastic artificial skin increased the amount of AD retained. Among component B formulations, B15 showed the highest AD retention at 121.15 µg/cm^2^ (25.24%), followed by B10 at 100.43 µg/cm^2^. For the synthesized elastic artificial skin, AB15 showed the highest AD retention at 112.27 µg/cm^2^ (23.39%) followed by AB10 at 103.39 µg/cm^2^. In addition, the results showed that the amount of AD retained from B15 and AB15 was approximately 2 and 4 times higher than that from B0 and AB0, respectively. This result might be attributed to SLNs because lipid-based nanoparticles exhibit effective permeation and retention in the intercellular lipids of the stratum corneum [77,91,93]. In this regard, after applying the elastic artificial skins to the skin, AD was consistently released from the reservoir of the intercellular lipids. In addition, such elastic artificial skins might help prevent water loss from the skin because of insufficient intercellular lipids in the stratum corneum were supplied [91,93].

## 4. Conclusions

The objective of this study was to prepare SLNs and elastic artificial skin using non-skin-irritating ingredients and improve the permeation of AD into the reconstructed human epidermal model. All components of SLNs and the elastic artificial skin were determined as non-skin-irritating substances. AD was successfully entrapped in SLNs using the modified double-emulsion method via a hot HPH technique. Evaluation of the loading capacity demonstrated that the higher the lipophilicity of the emulsion, the greater the amount of AD loaded in SLNs. In the elastic artificial skin containing SLNs, an increase in SLN concentration increased skin viscosity, thickness, EAB, and YM but decreased TS. The SLNs and elastic artificial skins exhibited sustained drug release profile in vitro. Particularly, the permeation study showed that all formulations improved the permeation of AD into the skin. Therefore, elastic artificial skin incorporated with AD-loaded SLN could serve as a highly promising topical drug delivery system for promoting AD permeation.

## Figures and Tables

**Table 1:** Composition of adenosine (AD)-loaded solid lipid nanoparticles.

Formulation	Drug (mg)	Lipid (g)					Lipophilic Surfactant (g)			Hydrophilic Surfactant (g)		
	AD	LUA	MA	PA	SA	GMS	SP			PX		TW
							80	40	20	188	407	80
F1	75	0.5					0.02			1		
F2	75		0.5				0.02			1		
F3	75			0.5			0.02			1		
F4	75				0.5		0.02			1		
F5	75					0.5	0.02			1		
F6	75				0.5			0.02		1		
F7	75				0.5				0.02	1		
F8	75				0.5		0.02				1	
F9	75				0.5		0.02					1
F10	25				0.5		0.02			1		
F11	50				0.5		0.02			1		
F12	75				0.5		0.1			1		
F13	75				0.5		0.5			1		
F14	75				1.5		0.06			1		
F15	75				1.5		0.3			1		
F16	75				1.5		1.5			1		
F17	75				2.5		0.1			1		
F18	75				2.5		0.5			1		
F19	75				2.5		2.5			1		

AD, adenosine; LUA, lauric acid; MA, myristic acid; PA, palmitic acid; SA, stearic acid; GMS, glycerol monostearate; SP 80, Span^®^ 80; SP 40, Span^®^ 40; SP 20, Span^®^ 20, PX 188, Poloxamer 188; PX 407, Poloxamer 407; TW 80, Tween^®^ 80.

**Table 2:** Variations in homogenization pressure and number of cycles assessed while fabricating solid lipid nanoparticles.

Pressure.		Number of Cycles	
Number of Cycles	Pressure (psi)	Pressure (psi)	Cycles
3	10,000	20,000	1
	15,000		2
	20,000		3
	25,000		4
	30,000		5

F4 was used to study the effect of high-pressure homogenization parameters.

**Table 3:** Composition of component A in the elastic artificial skin.

Ingredients	% Composition
HRC-LS-2830/1A	80
Serasense SF1	10
DC^®^ RM 2051	5
Cellulose nanofiber-graft-vinyltrimethoxysilane	5
Total	100

**Table 4:** Composition of component B in the elastic artificial skin.

Ingredients	% Composition
DI water	69.84
Edetate disodium	0.02
1,2-Hexanediol	2
1,3-Propanediol	5
Glycerin	5
M-Hydro EG	5
Sepimax Zen	0.5
Aristoflex AVC	0.1
HRC-LS-2830/1B	5
Olivem 1000	3
Olivem 900	1.5
Niacinamide	2
Adenosine	0.04
Sepiplus 400	1
Total	100

DI water, distilled water.

**Table 5:** Composition of the elastic artificial skin incorporated with adenosine-loaded solid lipid nanoparticles.

Formulation	Adenosine-Loaded Solid Lipid Nanoparticles (%)	B component of the Elastic Artificial Skin (%)
B0	0	100
B1	1	99
B5	5	95
B10	10	90
B15	15	85

**Table 6:** Tensile strength, elongation at break, and Young’s modulus of the elastic artificial skins with/without adenosine-loaded solid lipid nanoparticles.

Formulation	Tensile Strength (MPa)	Elongation at Break (%)	Young’s Modulus (MPa)
AB0	1.78 ± 0.15	12.00 ± 5.89	14.81 ± 1.28
AB1	1.90 ± 0.08	21.33 ± 3.40	8.93 ± 0.38
AB5	1.43 ± 0.09	35.33 ± 1.89	4.05 ± 0.24
AB10	1.11 ± 0.13	50.67 ± 2.49	2.19 ± 0.25
AB15	0.76 ± 0.14	58.67 ± 2.49	1.29 ± 0.24

Results are expressed as the means ± standard deviation of three independent experiments (*n* = 3).

## Data Availability

The data presented in this study are available in [Design and Characterization of Elastic Artificial Skin Containing Adenosine-Loaded Solid Lipid Nanoparticles for Treating Wrinkles].

## References

[1] Farahin A., Yusoff F., Basri M., Nagao N., Shariff M. (2019). Use of microalgae: Tetraselmis tetrathele extract in formulation of nanoemulsions for cosmeceutical application. J. Appl. Phycol..

[2] Manela-Azulay M., Bagatin E. (2009). Cosmeceuticals vitamins. Derm. Clin..

[3] Lupo M.P., Cole A.L. (2007). Cosmeceutical peptides. Dermatol. Ther..

[4] Pham Q.L., Jang H.J., Kim K.B. (2017). Anti-wrinkle effect of fermented black ginseng on human fibroblasts. Int. J. Mol. Med..

[5] Varani J., Spearman D., Perone P., Fligiel S.E., Datta S.C., Wang Z.Q., Shao Y., Kang S., Fisher G.J., Voorhees J.J. (2001). Inhibition of type I procollagen synthesis by damaged collagen in photoaged skin and by collagenase-degraded collagen in vitro. Am. J. Clin. Pathol..

[6] Chan E., Fernandez P., Merchant A., Montesinos M., Trzaska S., Desai A., Tung C., Khoa D., Pillinger M., Reiss A. (2006). Adenosine A2A receptors in diffuse dermal fibrosis: Pathogenic role in human dermal fibroblasts and in a murine model of scleroderma. Arthritis Rheum..

[7] Abella M. (2006). Evaluation of anti-wrinkle efficacy of adenosine-containing products using the FOITS technique. Int. J. Cosmet. Sci..

[8] Marin R.M., Franchini K.G., Rocco S.A. (2007). Analysis of adenosine by RP-HPLC method and its application to the study of adenosine kinase kinetics. J. Sep. Sci..

[9] Park J.-H., Allen M.G., Prausnitz M.R. (2006). Polymer microneedles for controlled-release drug delivery. Pharm. Res..

[10] Rouquette M., Lepetre-Mouelhi S., Couvreur P. (2019). Adenosine and lipids: A forced marriage or a love match?. Adv. Drug Deliv. Rev..

[11] Kazemzadeh-Narbat M., Annabi N., Tamayol A., Oklu R., Ghanem A., Khademhosseini A. (2015). Adenosine-associated delivery systems. J. Drug Target..

[12] Moser K., Kriwet K., Naik A., Kalia Y.N., Guy R.H. (2001). Passive skin penetration enhancement and its quantification in vitro. Eur. J. Pharm. Biopharm..

[13] Shah K.A., Date A.A., Joshi M.D., Patravale V.B. (2007). Solid lipid nanoparticles (SLN) of tretinoin: Potential in topical delivery. Int. J. Pharm..

[14] Gratieri T., Gelfuso G., Lopez R. (2008). Basic principles and applications of iontophoresis for cutaneous penetration of drugs. Quim. Nova.

[15] Charoo N.A., Rahman Z., Repka M.A., Murthy S.N. (2010). Electroporation: An avenue for transdermal drug delivery. Curr. Drug Deliv..

[16] Tesselaar E., Sjöberg F. (2011). Transdermal iontophoresis as an in-vivo technique for studying microvascular physiology. Microvasc. Res..

[17] Lopez R.F., Seto J.E., Blankschtein D., Langer R. (2011). Enhancing the transdermal delivery of rigid nanoparticles using the simultaneous application of ultrasound and sodium lauryl sulfate. Biomaterials.

[18] Kyung Oh E., Jin S.-E., Kim J.-K., Park J.-S., Park Y., Kim C.-K. (2011). Retained topical delivery of 5-aminolevulinic acid using cationic ultradeformable liposomes for photodynamic therapy. Eur. J. Pharm. Sci..

[19] Mishra S., Kesharwani R., Tiwari A., Patel D. (2016). Improvement of Drug Penetration through the Skin by Using Nanostructured Lipid Carriers (NLC). Int. J. Pharm. Pharm. Res..

[20] Marwah H., Garg T., Goyal A.K., Rath G. (2016). Permeation enhancer strategies in transdermal drug delivery. Drug Deliv..

[21] Tiwari S.B., Pai R.M., Udupa N. (2004). Influence of ultrasound on the percutaneous absorption of ketorolac tromethamine in vitro across rat skin. Drug Deliv..

[22] Garg T. (2016). Current nanotechnological approaches for an effective delivery of bio-active drug molecules in the treatment of acne. Artif. Cells. Nanomed. Biotechnol..

[23] Dhote V., Bhatnagar P., Mishra P.K., Mahajan S.C., Mishra D.K. (2012). Iontophoresis: A potential emergence of a transdermal drug delivery system. Sci. Pharm..

[24] Szebeni J., Baranyi L., Sávay S., Bodó M., Milosevits J., Alving C.R., Bünger R. (2006). Complement activation-related cardiac anaphylaxis in pigs: Role of C5a anaphylatoxin and adenosine in liposome-induced abnormalities in ECG and heart function. Am. J. Physiol. Heart Circ. Physiol..

[25] Ayari M.G., Kadhirvel P., Favetta P., Plano B., Dejous C., Carbonnier B., Agrofoglio L.A. (2019). Synthesis of imprinted hydrogel microbeads by inverse Pickering emulsion to controlled release of adenosine 5′-monophosphate. Mater. Sci. Eng. C.

[26] Swami R., Singh I., Jeengar M.K., Naidu V., Khan W., Sistla R. (2015). Adenosine conjugated lipidic nanoparticles for enhanced tumor targeting. Int. J. Pharm..

[27] Liu D., Chen L., Jiang S., Zhu S., Qian Y., Wang F., Li R., Xu Q. (2014). Formulation and characterization of hydrophilic drug diclofenac sodium-loaded solid lipid nanoparticles based on phospholipid complexes technology. J. Liposome Res..

[28] Ghadiri M., Fatemi S., Vatanara A., Doroud D., Najafabadi A.R., Darabi M., Rahimi A.A. (2012). Loading hydrophilic drug in solid lipid media as nanoparticles: Statistical modeling of entrapment efficiency and particle size. Int. J. Pharm..

[29] Gallarate M., Trotta M., Battaglia L., Chirio D. (2009). Preparation of solid lipid nanoparticles from W/O/W emulsions: Preliminary studies on insulin encapsulation. J. Microencapsul..

[30] Bhattacharjee A. (2013). Solid lipid nanoparticles technology as a novel platform for delivery of drugs. Indo. Am. J. Pharm. Res..

[31] Bikkad M.L., Nathani A.H., Mandlik S.K., Shrotriya S.N., Ranpise N.S. (2014). Halobetasol propionate-loaded solid lipid nanoparticles (SLN) for skin targeting by topical delivery. J. Liposome Res..

[32] Garcês A., Amaral M., Lobo J.S., Silva A. (2018). Formulations based on solid lipid nanoparticles (SLN) and nanostructured lipid carriers (NLC) for cutaneous use: A review. Eur. J. Pharm. Biopharm..

[33] Severino P., Silveira E.F., Loureiro K., Chaud M.V., Antonini D., Lancellotti M., Sarmento V.H., da Silva C.F., Santana M.H.A., Souto E.B. (2017). Antimicrobial activity of polymyxin-loaded solid lipid nanoparticles (PLX-SLN): Characterization of physicochemical properties and in vitro efficacy. Eur. J. Pharm. Biopharm..

[34] Teixeira M., Carbone C., Souto E. (2017). Beyond liposomes: Recent advances on lipid based nanostructures for poorly soluble/poorly permeable drug delivery. Prog. Lipid Res..

[35] Rao S., Tan A., Thomas N., Prestidge C.A. (2014). Perspective and potential of oral lipid-based delivery to optimize pharmacological therapies against cardiovascular diseases. J. Control. Release.

[36] Han Y., Hu J., Jiang L. (2018). Collagen skin, a water-sensitive shape memory material. J. Mater. Chem. B..

[37] Yeo J.C., Lim C.T. (2016). Emerging flexible and wearable physical sensing platforms for healthcare and biomedical applications. Microsyst. Nanoeng..

[38] Nicoletti G., Brenta F., Bleve M., Pellegatta T., Malovini A., Faga A., Perugini P. (2015). Long-term in vivo assessment of bioengineered skin substitutes: A clinical study. J. Tissue Eng. Regen. Med..

[39] Brohem C.A., da Silva Cardeal L.B., Tiago M., Soengas M.S., de Moraes Barros S.B., Maria-Engler S.S. (2011). Artificial skin in perspective: Concepts and applications. Pigment Cell Melanoma Res..

[40] Bhowmick S., Thanusha A., Kumar A., Scharnweber D., Rother S., Koul V. (2018). Nanofibrous artificial skin substitute composed of mPEG–PCL grafted gelatin/hyaluronan/chondroitin sulfate/sericin for 2 nd degree burn care: In vitro and in vivo study. RSC Adv..

[41] Ma Z., Li S., Wang H., Cheng W., Li Y., Pan L., Shi Y. (2019). Advanced electronic skin devices for healthcare applications. J. Mater. Chem. B.

[42] Gholipourmalekabadi M., Seifalian A.M., Urbanska A.M., Omrani M.D., Hardy J.G., Madjd Z., Hashemi S.M., Ghanbarian H., Brouki Milan P., Mozafari M. (2018). 3D protein-based bilayer artificial skin for the guided scarless healing of third-degree burn wounds in vivo. Biomacromolecules.

[43] Anjum S., Arora A., Alam M., Gupta B. (2016). Development of antimicrobial and scar preventive chitosan hydrogel wound dressings. Int. J. Pharm..

[44] Feula A., Tang X., Giannakopoulos I., Chippindale A.M., Hamley I.W., Greco F., Buckley C.P., Siviour C.R., Hayes W. (2016). An adhesive elastomeric supramolecular polyurethane healable at body temperature. Chem. Sci..

[45] Dąbrowska A., Rotaru G.M., Derler S., Spano F., Camenzind M., Annaheim S., Stämpfli R., Schmid M., Rossi R. (2016). Materials used to simulate physical properties of human skin. Ski. Res. Technol..

[46] Zou B., Chen Y., Liu Y., Xie R., Du Q., Zhang T., Shen Y., Zheng B., Li S., Wu J. (2019). Repurposed leather with sensing capabilities for multifunctional electronic skin. Adv. Sci..

[47] Wang J.N., Liu Y.Q., Zhang Y.L., Feng J., Wang H., Yu Y.H., Sun H.B. (2018). Wearable superhydrophobic elastomer skin with switchable wettability. Adv. Funct. Mater..

[48] Wang T., Zhang Y., Liu Q., Cheng W., Wang X., Pan L., Xu B., Xu H. (2018). A self-healable, highly stretchable, and solution processable conductive polymer composite for ultrasensitive strain and pressure sensing. Adv. Funct. Mater..

[49] Li P., Zhang A., Zhou S. (2019). One-component waterborne in vivo cross-linkable polysiloxane coatings for artificial skin. J. Biomed. Mater. Res. A.

[50] Lee K.E., Poh B.T., Morad N., Teng T.T. (2008). Synthesis and characterization of hydrophobically modified cationic acrylamide copolymer. Int. J. Polym. Anal. Charact..

[51] Nho Y.-C., Park J.-S., Lim Y.-M. (2014). Preparation of poly (acrylic acid) hydrogel by radiation crosslinking and its application for mucoadhesives. Polymers.

[52] Aziz T., Fan H., Khan F.U., Haroon M., Cheng L. (2019). Modified silicone oil types, mechanical properties and applications. Polym. Bull..

[53] Yu B., Kang S.-Y., Akthakul A., Ramadurai N., Pilkenton M., Patel A., Nashat A., Anderson D.G., Sakamoto F.H., Gilchrest B.A. (2016). An elastic second skin. Nat. Mater..

[54] Snorradottir B., Gudnason P., Scheving R., Thorsteinsson F., Masson M. (2009). Release of anti-inflammatory drugs from a silicone elastomer matrix system. Pharmazie.

[55] Alépée N., Tornier C., Robert C., Amsellem C., Roux M.-H., Doucet O., Pachot J., Méloni M., de Fraissinette A.d.B. (2010). A catch-up validation study on reconstructed human epidermis (SkinEthic™ RHE) for full replacement of the Draize skin irritation test. Toxicol. In Vitro.

[56] Odraska P., Mazurova E., Dolezalova L., Blaha L. (2011). In vitro evaluation of the permeation of cytotoxic drugs through reconstructed human epidermis and oral epithelium. Klin. Onkol..

[57] Schäfer-Korting M., Bock U., Diembeck W., Düsing H.-J., Gamer A., Haltner-Ukomadu E., Hoffmann C., Kaca M., Kamp H., Kersen S. (2008). The use of reconstructed human epidermis for skin absorption testing: Results of the validation study. Altern. Lab. Anim..

[58] Su N., An Q., Zhao D., Zheng H., Yang L., Li X., Wang C. (2017). Assessment and comparison of the skin irritation potential of five plant extracts using In Vitro and In Vivo methods. Am. J. Biomed. Sci..

[59] Hazirah M.N., Isa M., Sarbon N. (2016). Effect of xanthan gum on the physical and mechanical properties of gelatin-carboxymethyl cellulose film blends. Food Packag. Shelf Life.

[60] Schmid M. (2013). Properties of cast films made from different ratios of whey protein isolate, hydrolysed whey protein isolate and glycerol. Materials.

[61] Pritchard E.M., Szybala C., Boison D., Kaplan D.L. (2010). Silk fibroin encapsulated powder reservoirs for sustained release of adenosine. J. Control. Release.

[62] Mishra P.R., Al Shaal L., Müller R.H., Keck C.M. (2009). Production and characterization of Hesperetin nanosuspensions for dermal delivery. Int. J. Pharm..

[63] Chetoni P., Burgalassi S., Monti D., Tampucci S., Tullio V., Cuffini A.M., Muntoni E., Spagnolo R., Zara G.P., Cavalli R. (2016). Solid lipid nanoparticles as promising tool for intraocular tobramycin delivery: Pharmacokinetic studies on rabbits. Eur. J. Pharm. Biopharm..

[64] Pashkovskaya A.A., Vazdar M., Zimmermann L., Jovanovic O., Pohl P., Pohl E.E. (2018). Mechanism of long-chain free fatty acid protonation at the membrane-water interface. Biophys. J..

[65] Müller R.H., MaÈder K., Gohla S. (2000). Solid lipid nanoparticles (SLN) for controlled drug delivery–a review of the state of the art. Eur. J. Pharm. Biopharm..

[66] Kwon H.-Y., Lee J.-Y., Choi S.-W., Jang Y., Kim J.-H. (2001). Preparation of PLGA nanoparticles containing estrogen by emulsification–diffusion method. Colloids Surf. A Phys. Eng. Asp..

[67] Mehnert W., Mäder K. (2012). Solid lipid nanoparticles: Production, characterization and applications. Adv. Drug Deliv. Rev..

[68] Paquin P. (1999). Technological properties of high pressure homogenizers: The effect of fat globules, milk proteins, and polysaccharides. Int. Dairy J..

[69] Floury J., Desrumaux A., Lardières J. (2000). Effect of high-pressure homogenization on droplet size distributions and rheological properties of model oil-in-water emulsions. Innov. Food Sci. Emerg. Technol..

[70] Innocente N., Biasutti M., Venir E., Spaziani M., Marchesini G. (2009). Effect of high-pressure homogenization on droplet size distribution and rheological properties of ice cream mixes. J. Dairy Sci..

[71] Li Y., Wu C.-L., Liu J., Zhu Y., Zhang X.-Y., Jiang L.-Z., Qi B.-K., Zhang X.-N., Wang Z.-J., Teng F. (2018). Soy protein isolate-phosphatidylcholine nanoemulsions prepared using high-pressure homogenization. Nanomaterials.

[72] Solomando J., Antequera T., Ruiz-Carrascal J., Perez-Palacios T. (2020). Improvement of encapsulation and stability of EPA and DHA from monolayered and multilayered emulsions by high-pressure homogenization. J. Food Process. Preserv..

[73] Bhalekar M.R., Pokharkar V., Madgulkar A., Patil N., Patil N. (2009). Preparation and evaluation of miconazole nitrate-loaded solid lipid nanoparticles for topical delivery. AAPS PharmSciTech.

[74] Liu J., Hu W., Chen H., Ni Q., Xu H., Yang X. (2007). Isotretinoin-loaded solid lipid nanoparticles with skin targeting for topical delivery. Int. J. Pharm..

[75] Kale S.N., Deore S.L. (2017). Emulsion micro emulsion and nano emulsion: A review. Syst. Rev. Pharm..

[76] Raza K., Singh B., Lohan S., Sharma G., Negi P., Yachha Y., Katare O.P. (2013). Nano-lipoidal carriers of tretinoin with enhanced percutaneous absorption, photostability, biocompatibility and anti-psoriatic activity. Int. J. Pharm..

[77] Clares B., Calpena A.C., Parra A., Abrego G., Alvarado H., Fangueiro J.F., Souto E.B. (2014). Nanoemulsions (NEs), liposomes (LPs) and solid lipid nanoparticles (SLNs) for retinyl palmitate: Effect on skin permeation. Int. J. Pharm..

[78] Guaratini T., Gianeti M.D., Campos P.M. (2006). Stability of cosmetic formulations containing esters of Vitamins E and A: Chemical and physical aspects. Int. J. Pharm..

[79] Rao P.S., Kumar Rahul A. (2019). Effect of viscosity variation on non-Newtonian lubrication of squeeze film conical bearing having porous wall operating with Rabinowitsch fluid model. Proc. Inst. Mech. Eng. C. J. Mech. Eng. Sci..

[80] Singh U.P., Gupta R.S. (2012). Non-Newtonian effects on the squeeze film characteristics between a sphere and a flat plate: Rabinowitsch model. Adv. Tribol..

[81] Jridi M., Hajji S., Ayed H.B., Lassoued I., Mbarek A., Kammoun M., Souissi N., Nasri M. (2014). Physical, structural, antioxidant and antimicrobial properties of gelatin–chitosan composite edible films. Int. J. Biol. Macromol..

[82] Tong Q., Xiao Q., Lim L.-T. (2008). Preparation and properties of pullulan–alginate–carboxymethylcellulose blend films. Food Res. Int..

[83] Mitri K., Shegokar R., Gohla S., Anselmi C., Müller R.H. (2011). Lipid nanocarriers for dermal delivery of lutein: Preparation, characterization, stability and performance. Int. J. Pharm..

[84] Pardeike J., Hommoss A., Müller R.H. (2009). Lipid nanoparticles (SLN, NLC) in cosmetic and pharmaceutical dermal products. Int. J. Pharm..

[85] Gaur P.K., Mishra S., Bajpai M., Mishra A. (2014). Enhanced oral bioavailability of efavirenz by solid lipid nanoparticles: In vitro drug release and pharmacokinetics studies. Biomed. Res. Int.

[86] Zhong Q., Zhang L. (2019). Nanoparticles fabricated from bulk solid lipids: Preparation, properties, and potential food applications. Adv. Colloid Interface Sci..

[87] Garg A., Bhalala K., Tomar D.S. (2017). In-situ single pass intestinal permeability and pharmacokinetic study of developed Lumefantrine loaded solid lipid nanoparticles. Int. J. Pharm..

[88] Pardeike J., Weber S., Haber T., Wagner J., Zarfl H., Plank H., Zimmer A. (2011). Development of an itraconazole-loaded nanostructured lipid carrier (NLC) formulation for pulmonary application. Int. J. Pharm..

[89] Nahrup J.S., Gao Z., Mark J., Sakr A. (2004). Poly (dimethylsiloxane) coatings for controlled drug release—Polymer modifications. Int. J. Pharm..

[90] Prokopowicz M., Szewczyk A., Łunio R., Sawicki W. (2017). Monolithic polydimethylsiloxane-modified silica composites prepared by a low-temperature sol–gel micromolding technique for controlled drug release. React. Funct. Polym..

[91] Gönüllü Ü., Üner M., Yener G., Karaman E.F., Aydoğmuş Z. (2015). Formulation and characterization of solid lipid nanoparticles, nanostructured lipid carriers and nanoemulsion of lornoxicam for transdermal delivery. Acta. Pharm..

[92] Bhalekar M., Upadhaya P., Madgulkar A. (2017). Formulation and characterization of solid lipid nanoparticles for an anti-retroviral drug darunavir. Appl. Nanosci..

[93] Ramasamy T., Khandasami U.S., Ruttala H., Shanmugam S. (2012). Development of solid lipid nanoparticles enriched hydrogels for topical delivery of anti-fungal agent. Macromol. Res..

